# Targeting ubiquitin-specific protease 22 suppresses growth and metastasis of anaplastic thyroid carcinoma

**DOI:** 10.18632/oncotarget.9098

**Published:** 2016-04-29

**Authors:** Hua-Dong Zhao, Hai-Li Tang, Ning-Ning Liu, Ya-Li Zhao, Qin-Qin Liu, Xiao-Shan Zhu, Lin-Tao Jia, Chun-Fang Gao, An-Gang Yang, Jun-Tang Li

**Affiliations:** ^1^ Department of General Surgery, Tangdu Hospital, Fourth Military Medical University, Xi'an, Shaanxi 710032, China; ^2^ Centre of Inflammation and Cancer Research, 150th Central Hospital of PLA, Luoyang, Henan 471031, China; ^3^ State Key Laboratory of Cancer Biology, Department of Biochemistry and Molecular Biology, Fourth Military Medical University, Xi'an, Shaanxi 710032, China; ^4^ State Key Laboratory of Cancer Biology, Department of Immunology, Fourth Military Medical University, Xi'an, Shaanxi 710032, China

**Keywords:** anaplastic thyroid carcinoma, ubiquitin-specific protease 22, proliferation, invasion, apoptosis

## Abstract

Ubiquitin-specific protease 22 (USP22) aberrance has been implicated in several malignancies; however, whether USP22 plays a role in anaplastic thyroid carcinoma (ATC) remains unclear. Here, we report that USP22 expression is highly elevated in ATC tissues, which positively correlated with tumor size, extracapsular invasion, clinical stages, and poor prognosis of ATC patients. *In vitro* assays showed that USP22 depletion suppressed ATC cell survival and proliferation by decreasing Rb phosphorylation and cyclin D2, inactivating Akt, and simultaneously upregulating Rb; USP22 silencing restrained cell migration and invasion by inhibiting epithelial-mesenchymal transition; USP22 knockdown promoted mitochondrion- mediated and caspase-dependent apoptosis by upregulating Bax and Bid and promoting caspase-3 activation. Consistent with *in vitro* findings, downregulation of USP22 in ATC cells impeded tumor growth and lung metastasis *in vivo*. These results raise the applicability for USP22 as a useful predictor of ATC prognosis and a potential therapeutic target for ATC.

## INTRODUCTION

Anaplastic thyroid carcinoma (ATC) is very rare and aggressive in humans. Although ATC represents less than 2% of all thyroid cancers, it is responsible for more than 50% of thyroid cancer mortality, with 4–12 months of median survival time from diagnosis [1]. ATC has rapid growth and invasion-enabling capacities, reflected by marked expansion of tumor masses and outward invasion into neighboring organs [2]. To date, therapeutic options for ATC patients are limited and prognosis remains dismal [3]. Thus, identifying the efficient target to treat ATC is of urgency and critical clinical importance.

Ubiquitin-specific protease22 (USP22), is a novel deubiquitinating enzyme gene and belongs to a large family of proteins with ubiquitin hydrolase activity [4]. It was first identified as one of the cohort of genes that predict the recurrence, metastasis, and/or therapeutic responses of various types of cancers, known as the “death-from-cancer” signature. USP22 overexpression is detected in many human tumors, including non-small cell lung cancer, salivary duct carcinoma, bladder cancer, colorectal cancer, oral squamous cell carcinoma, and esophageal squamous cell carcinoma [5–11]. Elevated USP22 protein levels are associated with advanced tumor stage and poor prognosis in several cancer types [12, 13]. In cancer cells, recruited by the Myc oncoprotein or nuclear receptors, USP22 deubiquitylates histone H2A and H2B, and is necessary to counteract heterochromatin silencing and thereby transactivate specific target genes [14–16]. USP22 activation markedly contributes to aberrant cell cycle control and anoikis resistance and inhibits premature senescence [17]. USP22 promotes B-cell-specific murine leukemia virus integration site-1 (BMI-1)-induced epithelial-mesenchymal transition (EMT) [7, 12]. A previous study demonstrated that USP22 is highly expressed in papillary thyroid carcinoma and can be employed as an independent predictor of poor prognosis [18]. However, the expression and functions of USP22 in ATC remain largely elusive.

In this study, we investigated the role of USP22 in the development and progression of ATC. We found that upregulation of USP22 was well associated with tumor outgrowth, extracapsular invasion, clinical stages, and poor prognosis of ATC. USP22 depletion could significantly inhibit the proliferation and invasion, and promote the apoptosis of ATC cells *in vitro*. USP22 knockdown impaired ATC growth and lung metastasis *in vivo*. Hence, these data suggest that USP22 is a useful predictor of prognosis in ATC patients, and that the potential benefit of inhibiting USP22 is of putative clinical relevance in ATC treatment.

## RESULTS

### Overexpression of USP22 is associated with clinicopathological characteristics and prognosis of ATC

**Figure 1:**
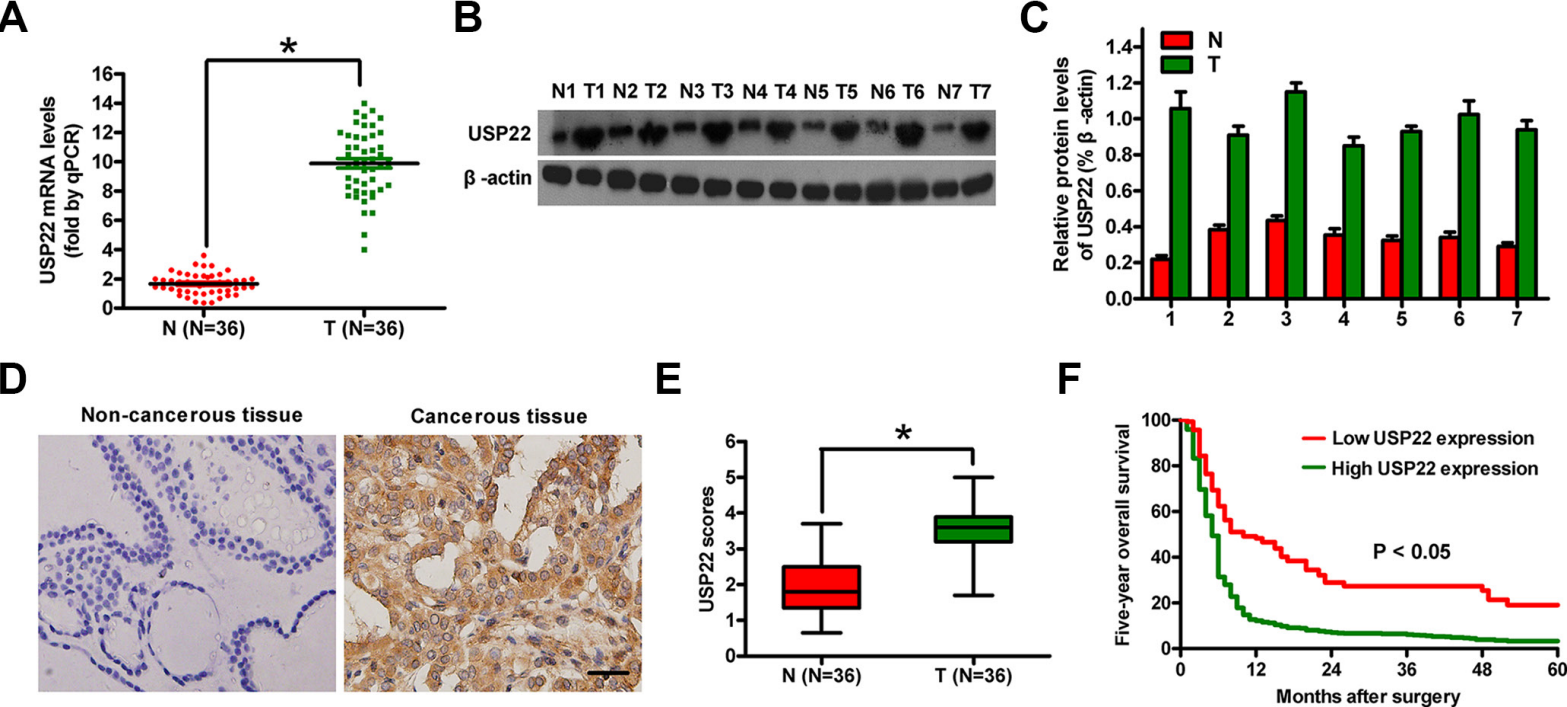
USP22 expression in ATC tissue samples and its association with prognosis (**A**) mRNA expression of USP22 analyzed by qPCR in 20 pairs of ATC (T) and adjacent non-cancerous (N) tissues. (**B**) Representative results of western blot analysis of USP22 expression; β-actin was used as endogenous control. (**C**) Quantification of USP22 protein in (B). (**D**) IHC analysis of USP22 protein expression in 36 T and matched N tissues. Representative photographs of USP22 staining. Scale bar: 10 μm. (**E**) USP22 expression scores in (D). (**F**) Relationship between USP22 protein expression by IHC and five-year overall survival rate after surgery in ATC patients (*n* = 36). Bar graph represented mean ± SD. Statistical significance: ^*^P < 0.05, as compared with N group (A, E).

We compared USP22 expression in clinical ATC (T) and non-cancerous (N) tissues. As shown in Figure 1A–1C, both mRNA and protein levels of USP22 were much higher in T tissues than N tissues. Immunohistochemistry assay also showed strong positive staining of USP22 in ATC specimens (Figure 1D and 1E). In addition, the five-year overall survival rate of patients with high USP22 expression was lower than those with low USP22 level (Figure 1F). As summarized in Supplementary Table S1, high USP22 level was positively associated with large tumor size, extracapsular invasion, lymph node metastasis, distant metastasis, and TNM stage, but not significantly related with gender and age. Thus, USP22 overexpression correlates with the occurrence, progression and poor prognosis of ATC.

### USP22 depletion inhibits the proliferation of ATC cells *in vitro*

**Figure 2:**
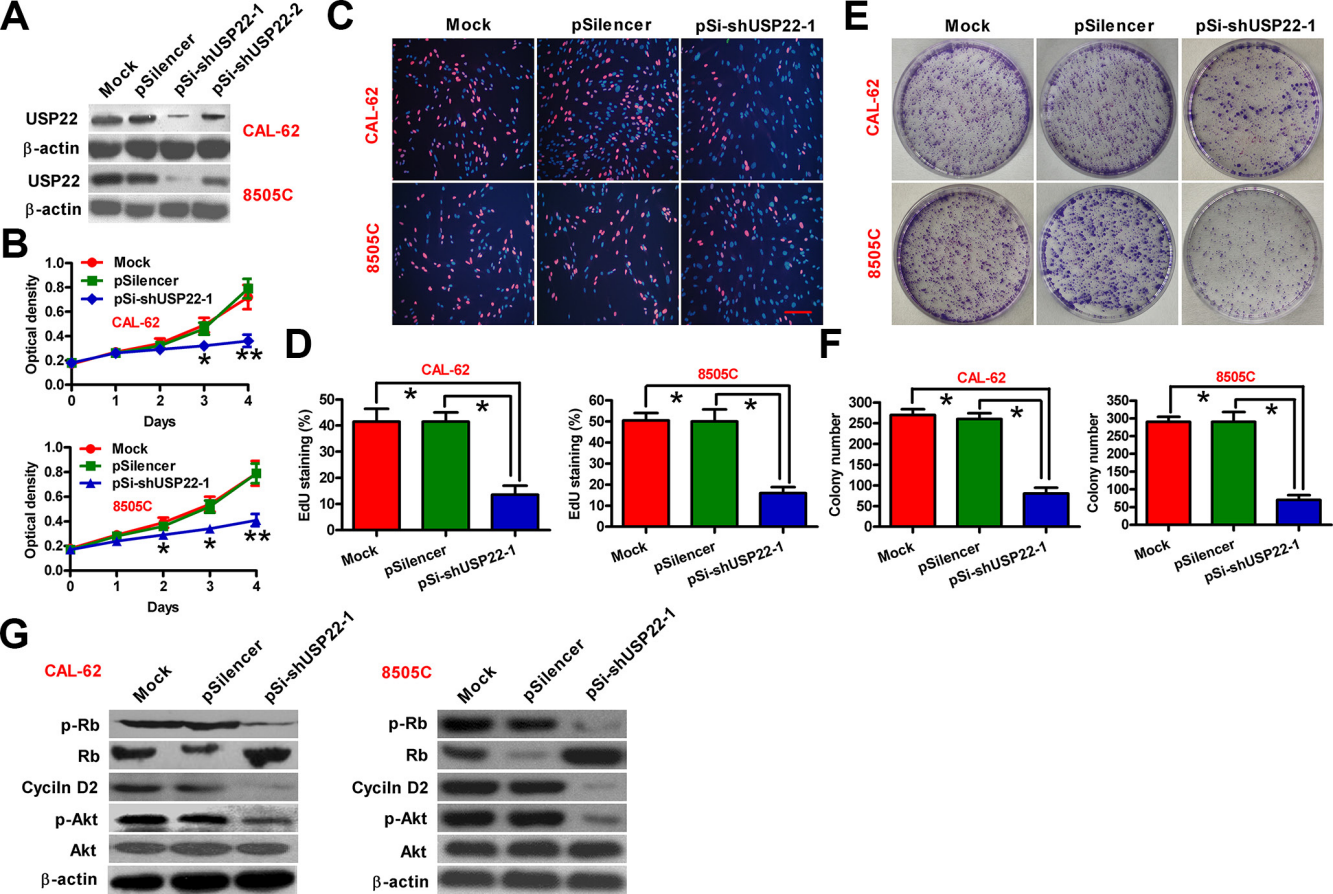
Inhibitory effect of USP22 depletion on ATC cell proliferation CAL-62 and 8505C cells were untransfected (Mock) or transfected with pSilencer, pSi-shUSP22-1 or pSi-shUSP22-2 plasmid. (**A**) Western blot analysis of USP22 expression; β-actin was used as endogenous control. (**B**) Cell viability was measured using MTT assay at 1, 2, 3, and 4 d after transfection. (**C**) EdU staining used to examine the proliferation. Scale bar: 5 μm. (**D**) Percentage of EdU-positive staining in (C). (**E**) Effect of USP22 knockdown on cell proliferation evaluated by colony formation assay. After 21 d of transfection, cells were stained with Giemsa solution. (**F**) Histograms representing the colony formation number in (E). (**G**) Western blot conducted to analyze the expression of USP22 and proliferation-related proteins including Rb, p-Rb, Akt, p-Akt, and cyclin D2. β-actin was used as endogenous control. The data were from three independent experiments. Bar graph represented mean ± SD. Statistical significance: ^*^P < 0.05, ^**^P < 0.01, as compared with mock or pSilencer group (B, D, and F).

To further evaluate the association of USP22 with ATC malignancies, we analyzed USP22 levels in ATC cell lines (CAL-62 and 8505C) and one benign human thyroid follicular cell line (Nthy-ori 3-1). Both mRNA and protein levels of USP22 were much higher in CAL-62 and 8505C cells than those in Nthy-ori 3-1 cells (Supplementary Figure S1A–S1C). Short hairpin RNA (shRNA) was used to knockdown USP22 in ATC cells (Figure 2A and Supplementary Figure S2). The shRNA construct that caused more potent inhibition of USP22 (pSi-shUSP22-1) was used in the subsequent experiments. Knockdown of USP22 significantly inhibited the viability of CAL-62 and 8505C cells (Figure 2B), and impaired proliferation as revealed by reduced EdU incorporation in CAL-62 and 8505C cells (Figure 2C and 2D). The *in vitro* colony formation capability of CAL-62 and 8505C cells was decreased in response to USP22 silencing (Figure 2E and 2F). Mechanistically, we found that USP22 depletion dramatically decreased Akt phosphorylation and cyclin D2 expression (Figure 2G). Interestingly, the retinoblastoma (Rb) protein, a negative regulator of cell cycle, was upregulated, and Rb activity was maintained due to decreased phosphorylation in USP22 knockdown cells (Figure 2G). Together, these data showed that USP22 silencing inhibited the proliferation of ATC cells *in vitro*.

### USP22 silencing suppresses the migration and invasion of ATC cells *in vitro*

**Figure 3:**
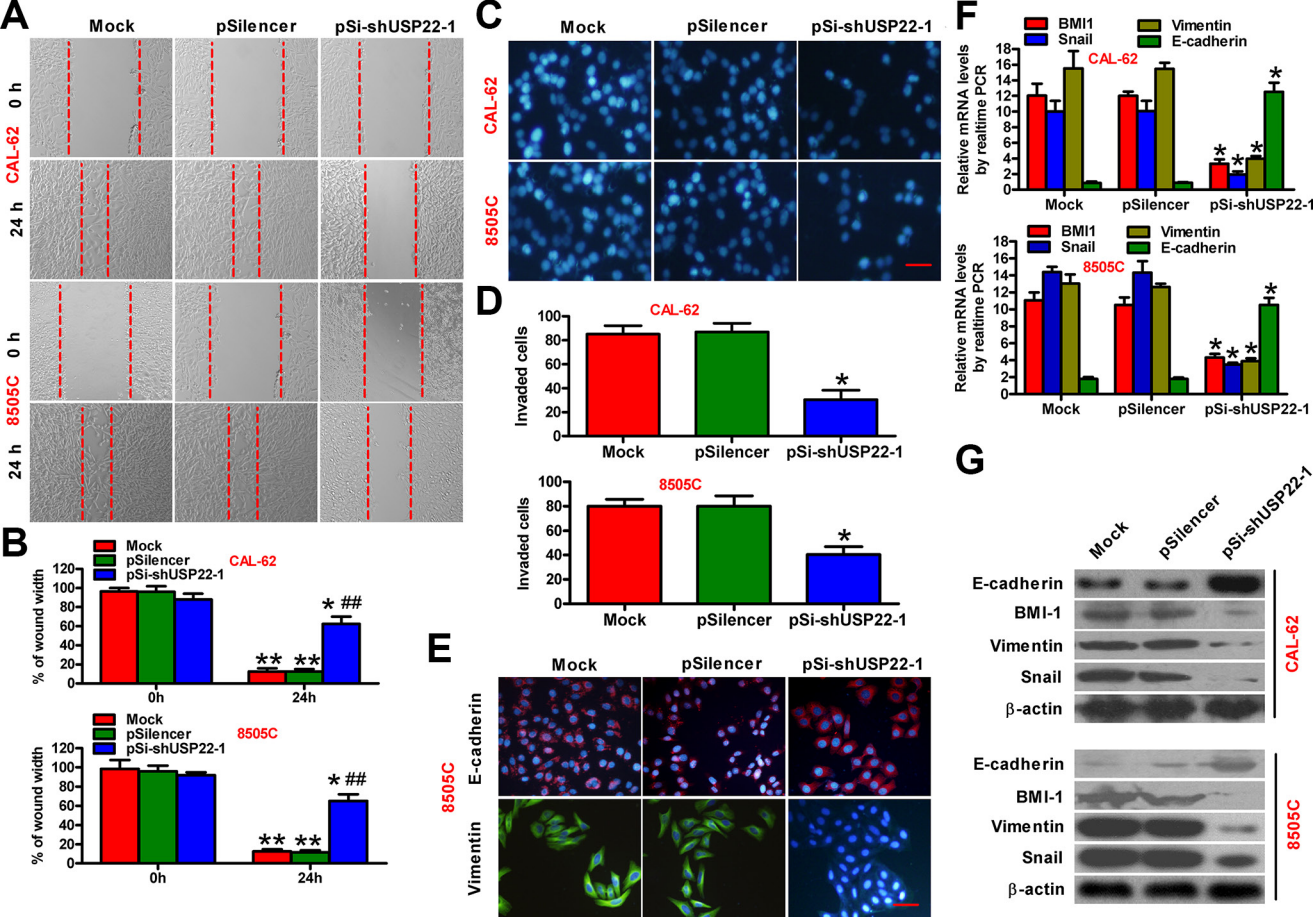
Knockdown of USP22 inhibits ATC cell migration and invasion CAL-62 and 8505C cells were untransfected (Mock) or transfected with pSilencer or pSi-shUSP22-1 plasmid. (**A** and **B**) After 48 h of transfection, the scratch wound-healing assay was performed to evaluate the effect of USP22 on cell migration. The representative images of cell migration were shown in (A), and the width of wounds at the indicated times was shown in (B). (**C**) Invasion of CAL-62 and 8505C cells analyzed by transwell assay. The invaded cells were stained with DAPI. Scale bar: 5 μm. (**D**) Number of DAPI-positive cells per field counted under a fluorescent microscopy. (**E**) Immunofluorescence staining was performed to analyze the effect of USP22 on the expression of E-cadherin and vimentin in 8505C cells. Scale bar: 5 μm. (**F** and **G**) Expression of invasion-related proteins containing BMI-1, E-cadherin, vimentin, and snail in ATC cells was measured by qPCR (F) and western blot (G). GAPDH and β-actin were used as endogenous controls, respectively. The data were from three independent experiments. Bar graph represented mean ± SD. Statistical significance: ^*^P < 0.05, ^**^P < 0.01, as compared with the group at 0 h after transfection (B). ^##^*P* < 0.01, as compared with mock or pSilencer group at 24 h after transfection (B). ^*^P < 0.05, as compared with mock or pSilencer group (D and F).

Migration and invasion of tumor cells through their basement membrane is an important process in the cascade of metastasis. We next investigated whether USP22 play a role in regulating the migration and invasion of ATC cells. Wound-healing assay showed that the mobility of CAL-62 and 8505C cells was evidently reduced after USP22 knockdown (Figure 3A and 3B). Similarly, knockdown of USP22 significantly reduced invasion of CAL-62 and 8505C cells in matrigel invasion assays (Figure 3C and 3D). The molecular mechanism of USP22 involving in tumor metastasis was next deciphered. Upon USP22 silencing, the epithelial cell marker E-cadherin was upregulated, whereas the expression of vimentin and snail, which was characteristic of mesenchymal cells, was significantly suppressed (Figure 3E–3G). In addition, the expression of BMI-1, a biomarker of cancer stem cells, was remarkably repressed in USP22 knockdown cells (Figure 3F and 3G). Therefore, USP22 silencing impairs ATC cell migration and invasion by ablating EMT.

### USP22 knockdown promotes apoptosis of ATC cells *in vitro*

**Figure 4:**
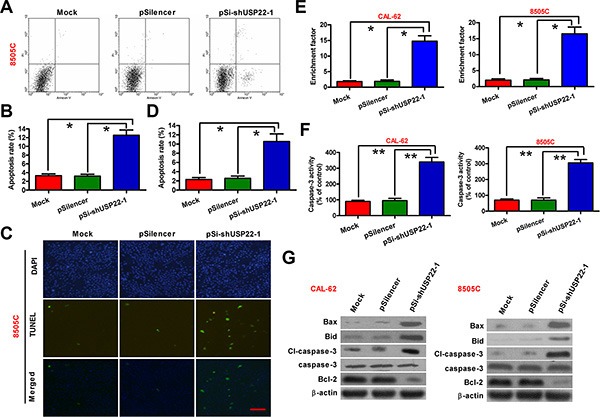
USP22 silencing promotes ATC cell apoptosis CAL-62 and 8505C cells were untransfected (Mock) or transfected with pSilencer or pSi-shUSP22-1 plasmid. (**A**) Apoptosis of 8505C cells analyzed by flow cytometry. (**B**) Apoptosis rate in (A) was calculated. (**C**) Fluorescent TUNEL assay conducted to determine the apoptosis of 8505C cells. Scale: 10 μm. (**D**) Percentage of TUNEL-positive cells in (C). (**E**) CAL-62 and 8505C cell apoptosis evaluated by nucleosomal fragmentation assay. (**F**) Quantification of caspase-3 activity in CAL-62 and 8505C cells. (**G**) Western blot analyses of apoptosis-related protein (Bid, Bax, cl-caspase-3, caspase-3, and Bcl-2) expressions in CAL-62 and 8505C cells. β-actin was used as endogenous control. The data were from three independent experiments. Bar graph represented mean ± SD. Statistical significance: ^*^P < 0.05, ^**^P < 0.01, as compared with mock or pSilencer group (B and D–F).

We next explored the role of USP22 depletion in ATC cell apoptosis. Flow cytometry analysis (Figure 4A and 4B) and TUNEL staining (Figure 4C and 4D) indicated that USP22 knockdown significantly increased the percentages of 8505C cells undergoing apoptosis. The nucleosomal enrichment factor assay also detected a significant increase in the ratios of apoptotic cells in CAL-62 and 8505C cells with USP22 knockdown (Figure 4E). To investigate the molecular mechanism underlying USP22 silencing-induced ATC cell apoptosis, we examined the cellular levels of documented apoptosis regulators or executioners. As a result, the activity of caspase-3 was markedly increased in USP22-depleted CAL-62 and 8505C cells (Figure 4F). The proapoptotic members of Bcl-2 family proteins, Bid and Bax, were upregulated, the processing and activation of procaspase-3 were increased, whereas the anti-apoptotic Bcl-2 was downregulated (Figure 4G). Overall, USP22 silencing could prompt ATC cell apoptosis *in vitro*.

### Effects of USP22 depletion on *in vivo* tumor growth and metastasis of ATC

**Figure 5:**
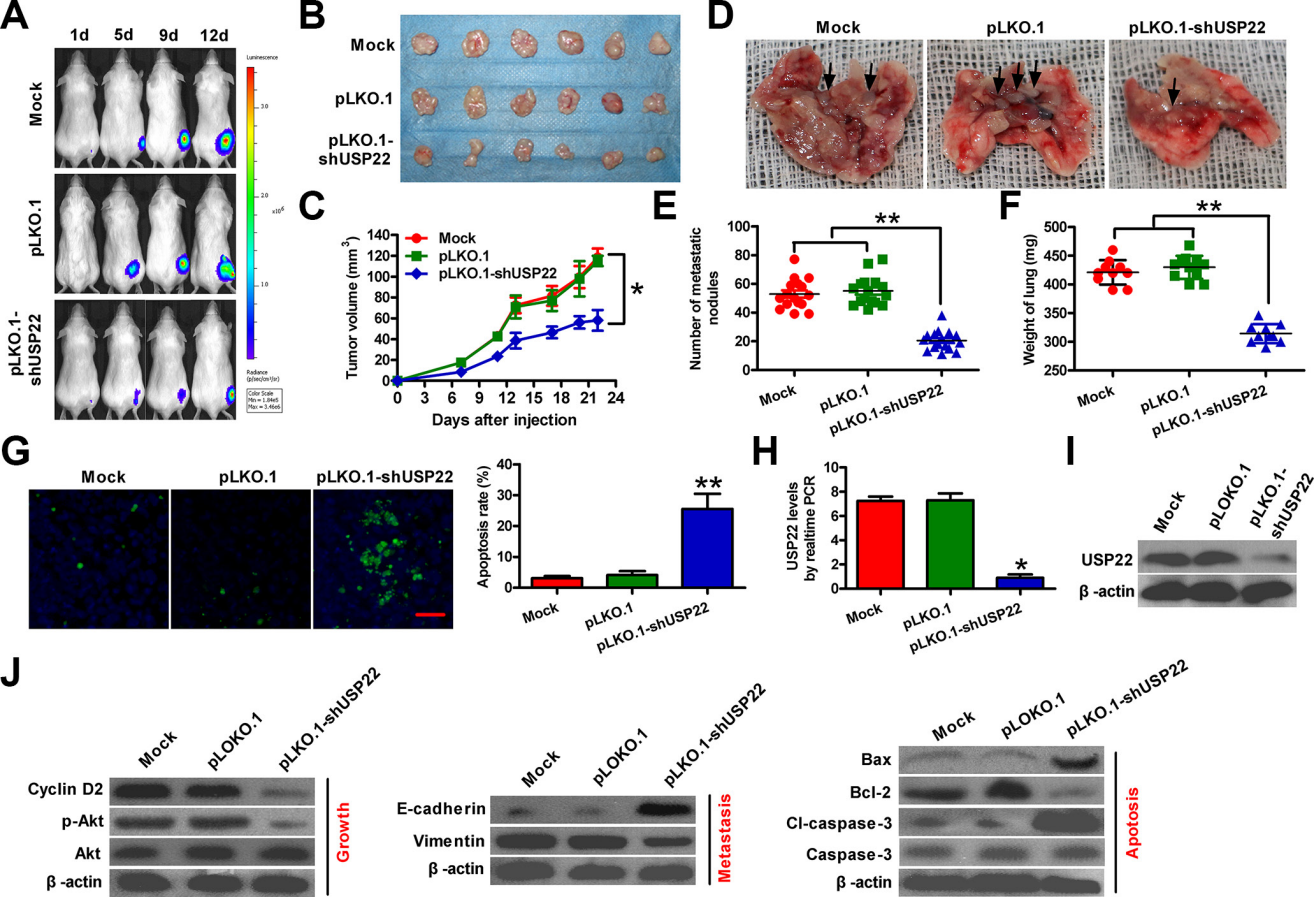
Knockdown of USP22 inhibits ATC tumorigenesis *in vivo* Female six-week-old SCID mice were inoculated subcutaneously into right hind flanks or injected via tail vein with stably expressed pLKO.1 or pLKO.1-shUSP22 8505C-luc cells. Mock-treatment was used as control. (**A**) Tumor growth progression was measured by *in vivo* luciferase imaging of the xenografts at days 1, 5, 9, and 12 d after inoculation. (**B**) Representative gross photos of tumors 22 d after subcutaneous xenografting (*n* = 6). (**C**) Tumor volumes of subcutaneous implantation models of ATC were monitored and calculated after 0, 7, 11, 13, 17, 20, and 22 d of inoculation. D–F. After 28 d of injection through tail vein, the lungs were removed and photographed (**D**), the number of metastatic nodules in lungs was counted (**E**), and the weight of lungs was measured (**F**). (**G**) Fluorescence TUNEL assay was carried out to determine cell apoptosis in the same tumor tissues as indicated above. The rate of TUNEL-positive cells was calculated. Scale bar: 10 μm. (**H**) qPCR assay was performed to detect the mRNA expression of USP22 in tumor tissues from (B). (**I**) Protein expression of USP22 in tumor tissues was analyzed by western blot. (**J**) Representative results of western blot analyses of cyclin D2, Akt, p-Akt, E-cadherin, vimentin, Bax, Bcl-2, cl-caspase-3, and caspase-3 in tumor tissues. (I and J) β-actin was used as endogenous control. The data were from three independent experiments. Bar graph represented mean ± SD. Statistical significance: ^*^P < 0.05, ^**^P < 0.01, as compared with mock or pLKO.1 group (C and E–H).

A xenograft model was developed using parental or USP22-silenced 8505C-luciferase (luc) cell line. Whereas palpable tumors formed one week after tumor cell implantation in both groups, the growth of tumors derived from USP22 knockdown 8505C-luc cells were compromised when compared with those from control 8505C-luc cells (Figure 5A and 5B). The volume of the tumors derived from 8505C-luc cells with USP22 depletion was lower than that of the tumors in the control groups (Figure 5C). Significantly lower frequency of lung metastasis and lung weight were detected in mice challenged intravenously (i.v.) with USP22-silenced 8505C-luc cells than in those receiving i.v. injection with parental cells (Figure 5D–5F). The number of TUNEL-positive cells markedly increased in USP22-depleted tumors compared to those with intact USP22 expression (Figure 5G). USP22 downregulation was observed in the tumor tissues from the mice with USP22-silenced 8505C-luc cells (Figure 5H and 5I). Molecular analysis of the tumor tissues showed that USP22 knockdown reduced the levels of cyclin D2, Akt phosphorylation, vimentin, and Bcl-2, whereas upregulated the expressions of E-cadherin, Bax, and cleaved (cl)-caspase-3, which confirmed *in vitro* findings. Collectively, these results suggested that USP22 depletion attenuates tumor growth and metastasis of ATC.

## DISCUSSION

ATC is a highly aggressive malignant cancer with a dismal prognosis [19]. Understanding the novel mechanisms of ATC development and identifying new targets to prevent ATC progression are the main challenges in the improvement of ATC treatment. Previous studies showed that USP22 silencing inhibits the progression of various types of tumors [7, 20, 21]. However, little is known with regards to the regulatory roles of USP22 in human ATC cells. Therefore, the present study aimed to determine the role of USP22 in ATC progression and its molecular mechanisms. We found that USP22 is frequently overexpressed in ATC tissues, positively associated with clinicopathological characteristics, and an independent prognostic indicator in ATC patients. Following USP22 silencing, the proliferation and invasion of ATC cells were markedly reduced, whereas ATC cell apoptosis was significantly increased. Consistently, USP22 knockdown resulted in suppressed tumor growth and metastasis of ATC *in vitro*.

Increasing evidences showed that USP22 serves a critical function in numerous pathological progresses and may be used as a highly promising diagnostic and/or prognostic marker of cancers [13, 17]. In this study, we demonstrated that USP22 expression was higher in ATC tissues than those in matched non-cancerous tissues, indicating its clinical significance. USP22 levels were positively associated with several key clinicopathological characteristics, suggesting that USP22 may serve an oncogenic function in promoting the progression of ATC, which adds further support to the concept that polycomb gene (PcG) pathway activation is mechanistically linked to the pathogenesis of solid tumors [22]. We also evaluated the therapy outcome-predictive power to further confirm the potential clinical utility of USP22. Kaplan–Meier analysis showed that the five-year overall survival rate of patients with high USP22 protein expression was significantly lower than that of patients with low USP22 protein levels. Therefore, USP22 may be a future diagnostic and/or prognostic marker of ATC patients.

USP22 has been shown to promote the proliferation of human non-small cell lung cancer cell H1129 and human bladder cancer cell EJ by facilitating cell cycle progression, which was supported by the observed G1 phase arrest and concomitant reduction in the S and G2/M phase when USP22 was depleted [7, 15]. Consistently, we demonstrated that USP22 silencing inhibited the proliferation of human ATC cells (8505C and CAL-62). Previous studies showed that Rb is a pivotal regulator in the G1 checkpoint of cell cycle [22, 23]. A delayed G1-to-S transition is usually accompanied by a reduction in the phosphorylation of Rb, which is indeed observed in USP22 knockdown ATC cells in our study, suggesting that USP22 may promote cell proliferation by modulating the Rb/E2F pathway [24]. Cyclin D2 is a very important early G1 phase cell cycle regulator and is essential in regulating Rb function in thyroid carcinogenesis [25]. Previous study also showed that USP22 promotes cell cycle progression by positively regulating the PI3K/Akt pathway [24]. Here, we showed that cyclin D2 expression and Akt activation were significantly suppressed upon USP22 silencing in ATC cells. These findings together with previous reports that PI3K/Akt signaling upregulates cyclin D2 via repression of the transcriptional factor FOXO1 [26] suggest that USP22 promotes cell cycle progression possibly via PI3K/Akt/cyclin D2 pathway in ATC cells.

It has been reported that USP22 silencing induced apoptosis of bladder [7], colorectal [20], and glioma [21] cancer cells. Consistently, in the current study, USP22 knockdown markedly induced ATC cell apoptosis, as evidenced by the results of flow cytometry, TUNEL, and nucleosomal enrichment factor assays. In the attempt to explore the mechanisms by which USP22 silencing leads to ATC cell apoptosis, we found that the proapoptotic members of Bcl-2 family proteins, Bid and Bax, were upregulated in response to USP22 knockdown, consistent with increased activation of caspase-3. Although it remains elusive whether USP22 downregulates Bid and Bax via direct transcriptional or posttranslational regulation, further investigations will help to determine whether the well characterized deubiquitinating enzyme activity of USP22 plays a role in the apoptotic machinery of ATC cells.

USP22 is positively associated with invasion and metastasis of multiple types of malignancies by mediating the oncoprotein BMI-1-driven pathways [7, 17, 24]. BMI-1 induced EMT and enhanced the invasion and metastasis of human nasopharyngeal epithelial cells and breast cancer cells, whereas BMI-1 silencing reduced cell motility and reversed EMT [27, 28]. EMT is known to be a central mechanism responsible for invasion and metastasis of various cancers, which endows the epithelial cells with mesenchymal-like properties, e.g. increased cell motility, and decreased intercellular adhesion [29, 30]. Downregulation of E-cadherin and upregulation of vimentin and snail are hallmarks of cells undergoing EMT [31]. In this study, we showed that USP22 depletion significantly decreased the expressions of BMI-1, vimentin, and snail and increased E-cadherin expression in ATC cells. In parallel, we achieved significantly suppressed lung metastasis formation by USP22 knockdown *in vivo*. These data suggest that USP22 serves as a critical regulator of ATC metastasis by promoting EMT. However, further investigations are needed to define the feedback mechanism underlying the maintenance of BMI-1 expression by USP22.

Although we detected USP22 expression and investigated the biological function of USP22 and related molecular events in ATC, this study had several limitations. Our statistical evidence may be insufficient because the present study encompassed a relatively small number of patients due to the rarity of ATC. Thus, the enrollment of a larger number of patients will be beneficial to corroborate the involvement of USP22-mediated pathways in the pathogenesis of clinical ATCs. Additionally, further investigation is required to elucidate whether USP22 is involved in other signaling pathways required for the progression of ATC.

In conclusion, the results presented here show that USP22 is upregulated in ATC and is positively correlated with tumor growth and metastasis. We also demonstrated that USP22 is an independent prognostic factor in ATC patients. The results of *in vitro* and *in vivo* studies confirmed USP22 depletion reduced the growth and invasion of ATC cells by regulating the expression and activation of a series of pro-tumorigenesis molecules. These results suggest that USP22 promotes tumor development and metastasis, and highlight USP22 as a novel prognostic marker and potential therapeutic target in ATC.

## MATERIALS AND METHODS

### Ethics statement

All experimental procedures were approved by the Institutional Review Board of the 150th Central Hospital of PLA and the Fourth Military Medical University. Written informed consent was obtained for all patient samples. Animal experiments were approved by the Institutional Committee for Animal Research and were performed in conformity with national guidelines for the care and use of laboratory animals.

### Clinical samples

For RNA extraction, the fresh 20 ATC and matched non-cancerous tissue (NCT) specimens were obtained from the patients who received surgical treatment without prior chemotherapy or radiotherapy in the 150th Central Hospital of PLA, or in Xijing Hospital of the Fourth Military Medical University and immediately frozen using liquid nitrogen and subsequently stored at −80°C for the following experiments. For immunohistochemistry (IHC), 36 formalin-fixed paraffin-embedded ATC and paired NCT specimens were collected from patients undergoing surgery without receiving chemotherapy or radiotherapy. All specimens were confirmed by pathological examinations. Postoperative pathological staging was determined by the tumor–node–metastasis (TNM) classification of the American Joint Committee on Cancer [32]. All patients were followed-up to 2015 or until death. The clinicopathological characteristics of the ATC patients are summarized in Supplementary Table S1. The Hospital Ethics Committee granted permission for the study.

### Cell culture

Human ATC cell lines CAL-62 and 8505C were purchased from Deutsche Sammlung von Mikroorganismen und Zellkulturen GmbH (DSMZ), which certifies the origin and identity of the cells. The benign human thyroid follicular cell line Nthy-ori 3–1 was obtained from American Type Culture Collection (Manassas, VA, USA). CAL-62 and 8505C were grown in Dulbecco's Modified Eagle Medium (DMEM; Sigma, St. Louis, MO, USA), and Nthy-ori 3–1 was maintained in Roswell Park Memorial Institute 1640 (Gibco, BRL, Grand Island, USA) with glutamine. All cell lines were used no later than 6 months after receipt and cultured with 10% fetal bovine serum (FBS; Gibco) and 1% penicillin/streptomycin (Invitrogen, Carlsbad, CA, USA) in an atmosphere of humidified air with 5% CO_2_ at 37°C.

### Establishment of 8505C-luc cell line

The lentivirus pLV-luc was purchased from Inovogen Biotechnology (Delhi, India) and infected 8505C cells. Cells were selected with puromycin (200 μg/mL; Sigma) to generate clones stably expressed luciferase. After 16 d screening, the single clone was obtained and was called 8505C-luc cell line.

### Transfection and infection

Transfection was performed with Lipofectamine 2000 (Invitrogen) according to the manufacturer's instructions. Plasmids pSilencer 3.1, pSi-shUSP22-1, and pSi-shUSP22-2 were obtained from GenePharma (Shanghai, China) and transfected into CAL-62 and 8505C cells. For plasmid transfections, 2 μg of DNA was used. Lentivirus pLKO.1 and pLKO.1-shUSP22 were purchased from GenePharma and infected 8505C cells. All constructs were confirmed by DNA sequencing. After 12 d of screening by 200 μg/mL neomycin (Sigma) for CAL-62 and 8505C cells transfected with pSilencer or pSi-shUSP22-1 plasmid (colony formation assay) or by 300 μg/mL puromycin (Sigma) for 8505C-luc cells infected with lentivirus pLKO.1 and pLKO.1-shUSP22 (*in vivo* assays), single clones were harvested. Transiently-transfected and stably-expressed ATC cells were obtained for the following experiments.

### RNA extraction and quantitative real-time PCR (qPCR)

Total RNA was extracted from 50 mg of tissue samples or 1 × 10^6^ cells using TRIzol reagent (Invitrogen) according to the manufacturer's protocol. The harvested RNA was diluted to a concentration of 1 μg/μL, packaged, and preserved at −80°C. cDNA was generated using the Reverse Transcription Kit (Promega, Madison, WI, USA). qPCR was performed using a standard IQTM SYBR Green Supermix kit (Bio-Rad, Berkeley, USA), and PCR-specific amplification was assessed by Mastercycler^®^ ep realplex (Eppendorf, Hamburg, Germany). GAPDH was used as endogenous control. The relative level of USP22 was calculated via the comparative 2^−ΔΔCt^ method [33]. Primer sequences were listed in Supplementary Table S2.

### Cell viability assay

Cell viability was measured using the 3-(4,5-dimethylthiazol-2-yl)-2, 5-diphenyl-tetrazolium bromide (MTT; Sigma) assay as described previously [34]. In brief, cells were seeded in a 96-well plate at a concentration of 1 × 10^3^ cells/well and cultured for 24 h until 50% to 60% confluence. After different treatments, cells were cultured for periods ranging from 1 d to 4 d. At the indicated time points, MTT (0.5 mg/mL) was added in each well and incubated at 37°C for 4 h. The supernatants were then removed, and 0.15 mL of dimethyl sulfoxide (Sigma) was added to each well; the plates were immediately read at 540 nm by using a scanning multi-well spectrometer (Bio-Tek instruments Inc., Winooski, VT, USA). All experiments were performed in triplicate.

### 5-ethyngl-20-deoxyuridine (EdU) incorporation assay

The cell proliferation was determined using EdU (RiboBio, Guangzhou, China) incorporation staining according to the manufacturer's instructions. Briefly, cells were treated with 50 μM EdU in medium in 6-well plates for 2 h, and then fixed by 4% paraformaldehyde for 20 min at room temperature. After washing twice with cold phosphate-buffed saline (PBS), six random fields were selected to observe and photograph under an inverted fluorescent microscope (Carl-Zeiss, Berlin, Germany).

### Colony formation assay

1% agar (Sigma) was melted in a microwave and transferred into a centrifugal tube to cool to 40°C in a water bath. The same volume of pre-warmed 2 × DMEM medium containing 20% FBS plus antibiotics (penicillin and streptomycin) was added into the tube and mixed for at least 30 min at 40°C for homogeneity. The prepared mixture was distributed into 6-well plates and set in a super-clean bench at room temperature for at least 1 h to allow agar to solidify completely. The pretreated cells at a final concentration of 2 × 10^3^ cells/well were seeded in the 6-well plate plus DMEM with 10% FBS. The 6-well plate was placed in an atmosphere of humidified air with 5% CO_2_ at 37°C. Fresh culture medium was replaced every 3 d. After 21 d, the clones were fixed with methanol and stained with 2% Giemsa solution (Merck, New York, USA). The colonies were visualized and counted under a microscope, as previously described [35]. All experiments were performed in triplicate.

### Wound-healing and invasion assay

For wound-healing assay, 1 × 10^6^ cells were seeded on 6 cm plates coated with 10 μg/mL type I collagen (Sigma). Cells were incubated for 24 h, and the monolayer was disrupted with a cell scraper, and photographs were taken at 0 and 24 h with a phase-contrast microscope (Olympus, Tokyo, Japan). For invasion assay, the transwell insert with an 8 μm diameter (Costar, Dallas, TX, USA) was coated with 200 μL matrigel (RD, Carlsbad, CA, USA) at 200 μg/mL and pre-incubated with DMEM medium. Cells were seeded into the upper chamber of the transwell (2 × 10^4^ cells/insert), and DMEM/10% FBS was added to the lower chamber. After 24 h of incubation at 37°C, cells were fixed in methanol and stained with 4′, 6-diamidino-2-phenylindole (DAPI; Sigma). Cells that invaded through the pores to the lower surface of the filter were counted under a microscope. Three invasion chambers were used per condition, and the total number of cells from the three filters was averaged.

### Nucleosomal fragmentation assay

Detection of apoptosis was fulfilled following the method described elsewhere [36]. After 48 h of mock treatment or transfection with pSilencer 3.1 or pSi-shUSP22-1, cell apoptosis was quantified by nucleosomal fragmentation (Cell Death Detection ELISA PLUS; Roche Applied Science, Indianapolis, IN, USA) according to the manufacturer's protocol. The absorbance values were normalized with reference to control-treated cells to derive a nucleosomal enrichment factor.

### Quantitative caspase-3 activity assay

Caspase-3 activity was detected using the Caspase-3/CPP32 Colorimetric Assay Kit (BioVision, Palo Alto, CA, USA) following the manufacturer's instructions. After 48 h of mock treatment or transfection with pSilencer 3.1 or pSi-shUSP22-1, 1 × 10^6^ cells were incubated with 50 μL of chilled lysis buffer on ice for 10 min. The supernatant was then collected after 10,000 × *g* centrifugation. Protein (150 μg) in a total volume of 50 μL was added to 50 μL 2 × reaction buffer containing 5 μL of N-Acetyl-Asp-Glu-Val-Asp-pNA substrate (200 μM final concentrations). After incubation at 37°C for 2 h, N-Acetyl-Asp-Glu-Val-Asp-pNA cleavage was monitored by detecting enzyme-catalyzed release of pNA at 405 nm using a microplate reader (Bio-Tek instruments Inc., Winooski, VT, USA).

### Apoptosis assay by flow cytometry

The apoptosis assay has been described elsewhere, using Annexin V-fluorescein isothiocyanate (FITC) and propidium iodide (PI; Beyotime, Haimen, China) staining [37]. After 48 h of mock treatment or transfection with pSilencer 3.1 or pSi-shUSP22-1, cells were harvested, centrifuged, and resuspended in binding buffer. Approximately 10 μL of ready-to-use Annexin V-FITC (BD Bioscience) was added in the mixture, incubated at 37°C for 15 min, and counterstained with 5 μL of PI in the dark for 30 min. Annexin V-FITC and PI fluorescence were assessed by BD FACSCalibur flow cytometry (BD Bioscience). Results were analyzed by CellQuest software (BD Bioscience). Each sample was prepared in triplicate.

### Terminal transferase-mediated dUTP nick end labeling (TUNEL) assay

TUNEL assay was used to detect DNA strand breaks *in situ* as previously described [38]. The untransfected or transfected cells seeded on glass slides were fixed with 80% glycerol at room temperature, rinsed with PBS (pH 7.4), and permeabilized with 2% Triton X-100. The FITC-labeled terminal deoxynucleotidyl transferase (TdT) nucleotide mix (Promega, Madison, WI, USA) was added to each slide and incubated at 37°C for 60 min. Slides were rinsed twice in PBS and counterstained with 10 mg/mL DAPI. FITC-labeled TdT was omitted in the nucleotide mix of the negative control. The tissue specimens were fixed with 10% formalin overnight, embedded with paraffin, non-serially sectioned (4 μm), and mounted on polylysine-covered slides. After deparaffinization in xylene and rehydration in a graded series of ethanol solutions, the sections were rinsed with PBS and incubated with FITC-labeled TdT nucleotide mix at 37°C for 60 min. Subsequently, the sections were rinsed twice in PBS and counterstained with 10 mg/mL DAPI. TUNEL-positive cells were imaged and mounted by fluorescence microscopy (Carl Zeiss) and ultimately expressed as a percentage of the total cells (DAPI staining).

### Immunofluorescence staining

The untransfected or transfected cells were grown on glass coverslips and were allowed to attach for 24 h prior to staining. The coverslips were washed, fixed in 3.7% formaldehyde, immersed sequentially in cold methanol and acetone, and then allowed to air-dry. The dry coverslips were incubated with diluted primary antibodies against E-cadherin (Abcam, Cambridge, UK) and vimentin (Abnova, Taiwan, China), and subsequently incubation with Cy3- or FITC-conjugated secondary antibody. The nuclei were counterstained with DAPI, and the images were captured using a fluorescence microscopy (Carl Zeiss).

### Western blotting

Proteins were extracted from fresh tissues and cells, separated by sodium dodecyl sulfate-polyacrylamide gel electrophoresis, transferred onto nitrocellulose membranes (Millipore, Bedford, MA, USA), and subjected to immunoblot analyses. Blotting was performed with primary antibodies targeting USP22, Akt, phosphorylated (p)-Akt (Ser473), Rb, and p-Rb (Ser811) (all from Cell Signaling Technology, Danvers, MA, USA), cyclin D2, BMI-1, and E-cadherin (all from Abcam, Cambridge, UK), vimentin, snail, Bid, Bax, and Bcl-2 (all from Abnova, Taiwan, China), cl-caspase-3, caspase-3, and β-actin (all from Sigma), followed by horseradish peroxidase-conjugated secondary antibody (Sigma). Bands were visualized using the enhanced chemiluminescence kit (Santa Cruz, Dallas, TX, USA). Protein band density was quantified using Quantity One software (Bio-Rad, Berkeley, CA, USA).

### Immunolohistochemistry and staining evaluation

All the tissue specimens were fixed with PBS-buffered 10% formalin overnight, embedded with paraffin, non-serially sectioned (4 μm), and mounted on the polylysine-covered slides. After deparaffinization in xylene and rehydration in a graded series of ethanol solutions, sections were submerged in citrate buffer (pH 6.0) and boiled in an autoclave at 121°C for 3 min to retrieve the antigenicity. The slides were then allowed to cool at room temperature. Endogenous peroxidase was quenched with 0.3% H_2_O_2_ in methanol for 15 min. Nonspecific adsorption was minimized by incubating the section in 10% normal goat serum (Gibco) in PBS for 20 min. Sections were incubated overnight with 1:150 dilution of primary anti-USP22 polyclonal antibody (Abcam) or with control solutions including buffer alone or nonspecific purified rabbit immunoglobin G (Sigma). Subsequently, sections were incubated with a biotinylated secondary antibody using the ChemMate EnVision Kit (DAKO, Hamburg, Germany) for 15 min. The reaction products were visualized with diaminobenzidine (DAB) substrate (Maixin Biotech., Fuzhou, China) as chromogen. The sections were counterstained with commercial hematoxylin (Maixin Biotech.), dehydrated, and mounted under light microscopy (Leica, Wetzlar, Germany). All stained sections were blindly observed and evaluated for DAB-positive staining by two experienced pathologists. Scores representing the extent (percentage of tumor cells stained positive) were as follows: 0, 0%; 1, 1% to 30%; 2, 31% to 60%; and 3, > 60%. Intensity was estimated and expressed as follows: 0, negative staining; 1, weak staining; 2, moderate staining; and 3, strong staining. The combination of the extent (*E*) and intensity (*I*) was obtained by the product of *E* × *I* called *EI*, which varied from 0 to 9 for each spot and was employed as the final staining score. According to the final scores, tumor tissues were divided into two following types: low-level USP22 group (with a score ≤ 3) and high-level USP22 group (with a score > 3).

### *In vivo* tumor growth, metastasis, and apoptosis assays

For tumor growth assays, female 6-week-old severe combined immune deficiency (SCID; Institute of Zoology, Chinese Academy of Sciences, Beijing, China) mice were injected subcutaneously into the right hind flank with 1 × 10^6^ 8505C-luc cells infected with control lentivirus or lentivirus expressing shUSP22 (*n* = 6 mice/group). After 0, 7, 11, 13, 17, 20, and 22 d of inoculation, tumor volumes were monitored and calculated as follows: tumor volume = width^2^ × length/2. All mice were sacrificed at 22 d post-inoculation, and tumors were removed and photographed.

For *in vivo* metastasis assays, female 6-week-old SCID mice were injected with 5 × 10^5^ 8505C-luc cells infected with control lentivirus or lentivirus expressing shUSP22 through the tail vein. The mice were monitored for general health status and evidence of morbidity related to the primary tumor or metastasis. Mice were sacrificed at 28 d post-injection and anatomized mice were examined for metastasis in the lung. The lungs were dissected from the mice and the weighted. Lungs with visible tumor colonies were fixed and embedded in paraffin, and three non-sequential sections per animal were obtained. The sections were stained with hematoxylin/eosin (Maixin Biotech.) and analyzed for the presence of metastasis by light microscopy. The total number of metastases per lung section was counted and averaged.

For apoptosis assays, the tissue sections were stained with TUNEL kits. TUNEL-positive cells were examined and mounted under fluorescence microscope.

### Bioluminescence imaging and quantification

Female 6-week-old SCID mice received 1 × 10^6^ 8505C-luc cells (in 100 μL of PBS) that were infected with control lentivirus or lentivirus expressing shUSP22 via subcutaneous injection. Tumor growth progression was measured by *in vivo* luciferase imaging of the xenografts at days 1, 5, 9, and 12 d after treatment. The *in vivo* luciferase imaging was performed by intraperitoneal injection of the mice with D-luciferin (Promega, Madison, WI, USA) at a dose of 150 mg/kg per mouse. The mice were anesthetized, and images were acquired using the Xenogen IVIS imaging system. The signals in defined regions of interest were quantified as photon flux (photons/s/cm^2^) using Living Image software (Xenogen Corporation, Berkeley, CA, USA).

### Statistical analysis

Data are expressed as means ± SD from three independent experiments. Data were analyzed by Student's *t* test and ANOVA, as indicated in the Figure legends. Correlations between USP22 expression and clinicopathological characteristics were analyzed by chi-square test and Fisher's exact test. Survival curves were plotted using the Kaplan–Meier method. The statistical significance between the cases with high and low USP22 expression was assessed using log-rank tests. All *P* values were two sided and obtained using the SPSS 13.0 software package (SPSS Inc., IL, USA). *P* < 0.05 was considered statistically significant.

## SUPPLEMENTARY MATERIALS FIGURES AND TABLES



## Abbreviations

ATC: anaplastic thyroid carcinoma
BMI-1: B-cell-specific murine leukemia virus integration site-1
cl-caspase-3: cleaved caspase-3
DAB: diaminobenzidine
DAPI: 4′ 6-diamidino-2-phenylindole
EdU: 5-ethynyl-2′-deoxyuridine
EMT: epithelial- mesenchymal transition
FBS: fetal bovine serum
FITC: fluorescein isothiocyanate
IHC: immunohistochemistry
MTT: 3-(4 5-dimethylthiazol-2-yl)-2 5-diphenyl- tetrazolium bromide
p-Akt: phosphorylated-Akt
PBS: phosphate-buffered saline
PI: propidium iodide
PI3K: phosphatidylinositol-3 kinase
p-Rb: phosphorylated-Rb
qPCR: quantitative real-time PCR
Rb: retinoblastoma
TdT: terminal deoxynucleotidyl transferase
TNM: tumor-node-metastasis
TUNEL: terminal transferase-mediated dUTP nick end labeling
USP22: ubiquitin-specific protease.
