# Supplementary material for: Targeting ubiquitin-specific protease 22 suppresses growth and metastasis of anaplastic thyroid carcinoma
Source: Oncotarget. 2016 Apr 29;7(21):31191–203. doi: 10.18632/oncotarget.9098 (PMC5058749; doi:10.18632/oncotarget.9098)
Supplement: Supplementary file 1 [file oncotarget-07-31191-s001.pdf]

# Targeting ubiquitin-specific protease 22 suppresses growth and metastasis of anaplastic thyroid carcinoma

## Supplementary Materials

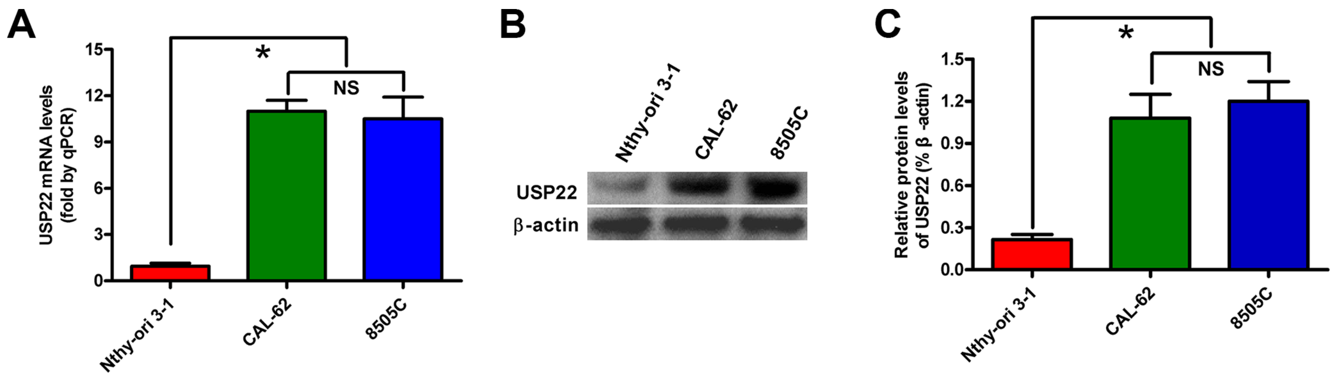


**Supplementary Figure S1: USP22 was upregulated in ATC cells.** (**A**) qPCR analysis of USP22 mRNA expression in benign human thyroid follicular cell (Nthy-ori 3–1) and ATC cells (CAL-62 and 8505C). GAPDH was used as the endogenous control. (**B**) USP22 protein expression was assessed by western blot in Nthy-ori 3-1, CAL-62 and 8505C cells. (**C**) Quantification of USP22 protein in (B). Bar graph represented mean ± SD. Statistical significance: $^{*}P < 0.05$, as compared with Nthy-ori 3–1 group.

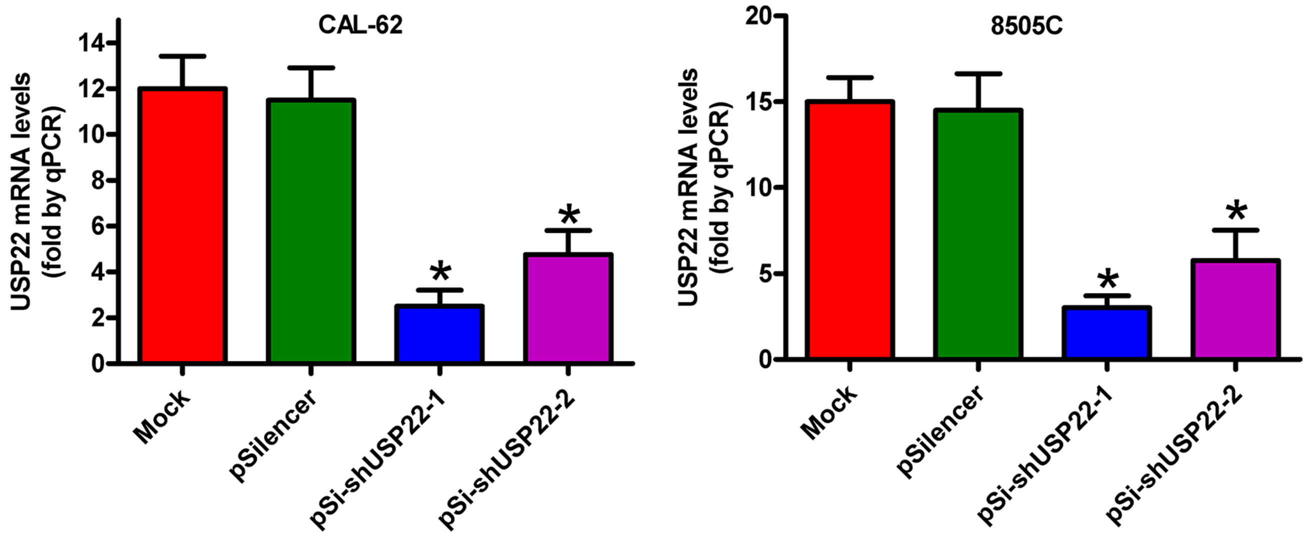


**Supplementary Figure S2: The knockdown efficiency of pSi-shUSP22 in ATC cells.** CAL-62 and 8505C cells were untransfected (Mock) or transfected with pSilencer, pSi-shUSP22-1 or pSi-shUSP22-2 plasmid. qPCR was performed to analyze USP22 mRNA expression. GAPDH was used as endogenous control. Bar graph represented mean ± SD. Statistical significance: $^{*}P < 0.05$, as compared with mock or pSilencer group.

**Supplementary Table S1: Association between USP22 expression and various clinicopathological characteristics of patients with ATC**

| Variables | Clinicopathological characteristics | Cases ($n$ = 36) | USP22 protein expression | | *P* value |
|---|---|---|---|---|---|
| | | | Low ($n$ = 15) | High ($n$ = 21) | |
| Gender | Male | 14 | 6 | 8 | 0.086 |
| | Female | 22 | 9 | 13 | |
| Age (years) | < 70 | 13 | 5 | 8 | 0.068 |
| | ≥ 70 | 23 | 10 | 13 | |
| Tumor size (cm) | ≤ 5 | 11 | 5 | 6 | 0.047* |
| | > 5 | 25 | 10 | 15 | |
| Extracapsular invasion | No | 10 | 6 | 4 | 0.032* |
| | Yes | 26 | 9 | 17 | |
| Lymph node metastasis | No | 14 | 8 | 6 | 0.015* |
| | Yes | 22 | 7 | 15 | |
| Distant metastasis | No | 9 | 7 | 2 | 0.001* |
| | Yes | 27 | 8 | 19 | |
| TNM stage | IVA | 4 | 1 | 3 | 0.029* |
| | IVB | 20 | 9 | 11 | |
| | IVC | 12 | 4 | 8 | |

*P* value represents the probability from a chi-square test for tissue USP22 levels in variable subgroups, $^*P < 0.05$.

## Supplementary Table S2: Primers used for qPCR

| Gene | Primer sequences (5′ to 3′) | |
|---|---|---|
| USP22 | Forward | GCTGCATTCCTGCCTCTA |
| | Reverse | GCTGCATTCCTGCCTCTA |
| BMI-1 | Forward | TGTAAAACGTGTATTGTTCGTTACC |
| | Reverse | CAATATCTTGGAGAGTTTTATCTGACC |
| Snail | Forward | GCTGCCAATGCTCATCTGGGACTCT |
| | Reverse | TTGAAGGGCTTTCGAGCCTGGAGAT |
| Vimentin | Forward | AAAGTGTGGCTGCCAAGAAC |
| | Reverse | GAGAGGTCAGCAA |
| E-cadherin | Forward | AGTCACGCTGAATACAGTGG |
| | Reverse | CATTTTCTGGGCAGCTGATG |
| GAPDH | Forward | GGAAATCGTGCGTGACATT |
| | Reverse | CAGGCAGCTCGTAGCTCTT |
